# Supporting perinatal anxiety in the digital age; a qualitative exploration of stressors and support strategies

**DOI:** 10.1186/s12884-020-02990-0

**Published:** 2020-06-17

**Authors:** Virginia Harrison, Donna Moore, Lisa Lazard

**Affiliations:** School of Psychology and Counselling, Faculty of Arts and Social Sciences, Open University, Milton Keynes, MK7 6AA UK

**Keywords:** Perinatal, anxiety, Female, postpartum, Mood disorder

## Abstract

**Background:**

The period surrounding childbirth is one of profound change, which can often be experienced as stressful and overwhelming. Indeed, around 20% of women may experience significant levels of anxiety in the perinatal period. However, most women experiencing perinatal anxiety (PNA) go unrecognised and untreated. The Internet offers a potentially scalable solution to improve access to support, however a dearth of research in this area means that work is needed to better understand women’s experience of PNA, so that potential targets for intervention can be identified and possible barriers to support overcome. This study aimed to qualitatively explore women’s experience of anxiety triggers and support in the perinatal period; and gain insight into what online support is acceptable for women with PNA.

**Methods:**

Women who were either pregnant or within one-year postpartum were invited to participate in focus groups across the UK. Focus groups were used to allow a diversity of perspectives to be heard, while simultaneously promoting the identification and prioritisation of important support needs and solutions. Interviews were transcribed and thematically analysed.

**Results:**

Five key themes emerged in relation to women’s experience with PNA: holding unrealistic expectations of birth and motherhood; stigma; the importance of peer support; uncertainty and poor maternal confidence; and a lack of mental health support and knowledge. Perinatal women felt under-supported and poorly prepared for motherhood. A mismatch between their expectations and the reality of their experience, alongside a pressure to be the ‘perfect mum’ was the primary source of their anxiety. Furthermore, stigma associated with PNA may have exacerbated these issues and led to help-seeking avoidance. Overall, women felt these issues could be addressed via online support, through the delivery of more realistic information, providing psychoeducation about PNA symptoms and management, and the inclusion of authentic peer experiences. Thus, delivering evidence-based information and interventions online may provide a solution that is acceptable to this cohort.

**Conclusions:**

This work provides unique insight into potential sources of anxiety for women in the perinatal period, while also offering potential internet-based support solutions that are likely to be acceptable and helpful for women with PNA.

## Background

Childbirth represents a significant period of physiological, behavioural and psychological change for women, which brings with it a risk of mental health problems, such as anxiety and depression [1–6]. Decades of research exists into the experience and treatment of antenatal and postnatal depression (PND), whereas perinatal anxiety (PNA) has received comparatively little attention. This is despite recent evidence that anxiety may occur at a higher rate and independently of PND [7–11].

Despite being treatable, few women experiencing PNA will seek help or receive efficacious treatment [12–14]. This is concerning, as research suggests untreated PNA may be associated with a variety of long- and short-term consequences in both the mother and infant, including preterm delivery, low birth weight, PND, excessive infant crying, bonding issues, problematic feeding behaviours, and adverse developmental, behavioural and emotional problems in children [9, 15–18]. Therefore, understanding the barriers to help-seeking and treatment access is crucial when considering how best to support women with PNA.

While there is little research into this in the context of PNA, the PND literature suggests key barriers to help-seeking include poor mental health literacy [19] and stigma [20–22]. Furthermore, as PNA is a relatively new concept, it is still poorly understood by the public and healthcare professionals (HCPs) alike. As such, women and HCPs may not recognise PNA symptoms, particularly as there are currently no PNA-specific screening tools used in routine clinical practice, and current diagnostic manuals (such as the Diagnostic and Statistical Manual of Mental Disorders, [23]) do not recognise PNA as a distinct disorder. This is problematic, as a significant proportion of perinatal women who experience significant anxiety symptoms do not meet the criteria for anxiety diagnosis [24, 25] and diagnosis is often a prerequisite for accessing support. Thus, support avenues that are independent of diagnosis should be investigated to see if they are acceptable to this cohort.

The internet is one such avenue of support that may be a valuable source of support for women experiencing PNA. It is low cost, available 24/7 and offers a level of anonymity that might circumvent stigma barriers [26]. Furthermore, online delivery offers scalable strategies for mental health care that may be useful in the treatment of mental health disorders, but also offer the opportunity for people with lower (or sub-clinical) levels of symptoms to get the help they need before full-syndrome disorder is realised. Given the long-term cost of PNA in the UK is estimated to be about £35 k per case (and significantly more when considering comorbid illness [27];), eHealth strategies that may be able to prevent the development of severe symptoms warrant investigation.

Over the last decade, a significant number of eHealth programs have been developed for mental health conditions. These programs have good acceptance and attitudes towards their use are positive in the general population [28]. Additionally, outcome data suggest that eMental Health programs are cost-effective and clinically efficacious, with effect sizes similar to face-to-face therapy [29–31]. Furthermore, previous research has shown that women with PNA would be interested in web-based PNA support [32].

While there are thousands of websites dedicated to perinatal mental health, they are predominantly aimed at PND [33]. A recent review of PNA websites found that they were often inaccurate, confusing and incomplete (frequently conflating PNA with PND), had limited availability and scope, and generally failed to offer direct support, which is unlikely to be helpful to PNA sufferers [34]. There is therefore a need for a gold-standard PNA-specific online resource. And while there is some preliminary evidence supporting the utility and attractiveness of eHealth programs in postnatal depression [35, 36], only one web-based PNA-specific intervention (iWaWa) has been evaluated to date [37] and it is not currently available for public use. While users generally considered this program to be helpful, adherence and engagement was low, and participants felt the content needed to be more inclusive and balanced. As such, the authors concluded it was not a feasible intervention in its current format, thus more work is needed to explore what women with PNA would find most helpful and engaging in terms of supporting their wellbeing online.

A bottom-up approach to developing an online supportive resource is likely to be helpful in terms of identifying potential targets for intervention, and maps onto recommendations about patient group involvement in the design of digital health programs/websites [38]. This notion is grounded in the assumption that women with PNA are ‘experts by experience’, who are likely to provide useful information about potential stressors and give insight into possible avenues of support. However, there is a dearth of qualitative research around women’s experience of PNA, particularly regarding triggers and support/coping mechanisms.

While some studies have focused on women’s experience of general psychological distress around childbirth [39–41], which has inevitably included some women experiencing anxiety, only two studies have employed qualitative methods to explore PNA specifically. Fallon et al. [42] carried out interviews with 19 post-partum UK mothers to generate a better understanding of women’s symptoms of PNA. However, this was done with the aim of creating a new PNA measurement scale so the qualitative information reported in this study is limited (although critical in terms of being able to better identify PNA). Wardrop and Popadiuk [43] interviewed 6 postpartum women to explore their experiences PNA. While the in-depth nature of these narrative interviews meant that rich data was collected about each woman’s experience, they were limited in number and focus. Discussion of PNA was limited to the first 6 months postpartum, however participants were able to take part up to 3 years after childbirth, bringing into question the clarity and representativeness of their accounts. Furthermore, while both of these studies were designed to capture women’s experience of PNA in some way, they did not take a solution-focused approach; and neither considered online contexts.

The aim of the current study is to use a qualitative approach to explore women’s experience of PNA, in particular, considering the main sources of their anxiety (i.e. triggers) and the support/coping strategies they use in both online and offline contexts. By exploring these issues, potential targets for intervention may be identified, and outcomes may provide a useful starting point for the development of an online support program. Thus, the aim of this study is twofold. First, to qualitatively explore women’s experience of anxiety triggers and support in the perinatal period; and second to gain insight into what online support is acceptable for women with PNA.

## Methods

### Design

Focus Groups (FGs) are widely used in health research to explore the perspectives of specific patient groups [44]. They can be more useful than individual interviews when the topic being explored is solution-focused, allowing for a diversity of opinions and experience to be discussed, while at the same time allowing for the identification, clarification and prioritisation of important topics and solutions [45]. Thus they are a useful method to develop knowledge about patient/participant experience, identify important support needs and aid the development of future intervention programs [46].

### Participants

Convenience sampling was used to recruit participants through advertising on social media and relevant forums (e.g. MumsNet), and via local mum and baby groups. Inclusion criteria were that women [1] were pregnant or had had a baby in the last 12 months; [2] were aged 20–45 years; [3] lived in the UK, [4] were fluent in English; and [5] had experienced anxiety during the perinatal period. Participants who expressed an interest were directed to the study website, which gave them more information about the study and obtained consent. Participants were then directed to a short, anonymous screening survey which assessed their suitability in terms of the inclusion criteria. To identify women who had experienced anxiety perinatally, we asked participants (1) how they rated their experience of anxiety from 1 to 5 (none, mild, moderate, severe, extremely severe); and (2) how much this distressed them on a scale of 1–5 (1 = not at all; 5 = severely). Participants who answered “none” on the initial anxiety scale were excluded from the study. This self-identification method was used to allow for the inclusion of participants with a broad range of PNA, including women with subsyndromal anxiety, as there is evidence that those who do not fulfil diagnostic criteria may still report high levels of distress and disability [47]. Participants had a mean anxiety score of 2.65 (SD = .65) and distress score of 2.52 (SD = .59).

Fifty-three participants expressed an interest in the study; 42 met eligibility criteria; and 23 were able to attend the times and locations available for the groups. Table 1 shows the demographic information for each group.

**Table 1:** Overview of participant characteristics (Ns)

FG	Area	Age	Ethnicity	Stage
1	Urban	25-30 yrs. = 1 31-35 yrs. = 2 36-40 yrs. = 3	White British = 3 White New Zealander = 1 White European = 2	Postpartum (primiparous) = 6
2	Rural	25-30 yrs. = 1 31-35 yrs. = 1 36-40 yrs. = 1	White British = 2 White & Black African = 1	Pregnant (multiparous) = 1 Postpartum (multiparous) = 2
3	Rural	31-35 yrs. = 1 36-40 yrs. = 1	White British = 2	Pregnant (multiparous) = 1 Postpartum (multiparous) = 1
4	Urban	25-30 yrs. = 1 31-35 yrs. = 4 36-40 yrs. = 1	White British = 5 White European = 1	Postpartum (primiparous) = 5 Postpartum (multiparous) = 1
5	Urban	31-35 yrs. = 1 36-40 yrs. = 4 41-45 yrs. = 1	White British = 4 Black African = 1 White New Zealander = 1	Postpartum (primiparous) = 6

### Procedure

FGs were conducted between May – July 2018, and were facilitated by VH and DM, both female lecturers at the Open University. In most cases, women attended with their babies. Semi-structured interview schedules (developed for this study, and found in Additional file 1) guided FG discussions, allowing for flexibility and insightful digressions. In the first half of the interview, participants were given some background information about the study and the interviewers' motivations for the research (namely that both interviewers have experience of PNA, and that it is an under-researched and under-resourced area), and were then invited to introduce themselves and say a bit about their experiences with pregnancy and/or motherhood. This involved an exploration of possible sources of anxiety during the perinatal period. The second half of the discussion focused on methods of support. Participants were asked to describe their experience of support for PNA, including the best sources of help and information. The final questions focused specifically on online support: asking about their experience with apps/websites to support mental wellbeing, their particular likes/dislikes of these apps/websites, and what the most important features of an online support program for PNA should be.

Each FG lasted approximately 60 min, was audio-recorded, transcribed verbatim, and checked by VH. As it was hypothesised that the support needs and choices of women may vary as a product of geography, five focus groups were held in community centres in different geographical locations around the UK: 3 in urban settings (different boroughs of London); and 2 in a rural area of Sussex.

### Data analysis

Data were analysed using inductive thematic analysis from a realist stance, as outlined by Braun and Clarke [48]. Initial codes were generated by VH and DM. To achieve this, transcripts were read several times to become familiar with the participants’ words and experiences. Next, within-transcript codes were generated (where possible) by using key words and phrases from the participants themselves, to keep their experiences at the heart of the analysis. These codes were then explored to identify initial patterns and themes that arose within each individual FG. This was done to ensure the codes that emerged retained their original meaning within the context of each FG. Once all transcripts had been coded, initial codes were pooled, and data reduction took place to group codes into coherent categories. Code categories were then reviewed, defined and indexed into subthemes and themes by VH. During this process, comparisons were continuously made across the transcripts to establish the relative importance of the emergent themes. To maximise reliability and to reduce bias in the analytic process, all three authors regularly discussed the coding and analytic procedure, alongside the interpretation of themes. If ambiguity arose, any differences were debated until a consensus was reached. Throughout the coding process, all authors used the techniques of bracketing and self-reflection in an attempt to keep participants’ experience at the centre of the codes and themes. This meant that authors gave priority to the interviewees’ accounts rather than their own personal or professional knowledge or experiences of PNA, and allowed them to reflect on how their own experiences might influence the analysis. To further validate the findings, an outline of the final themes derived was emailed to participants for checking.

## Results

Two main superorder themes were identified which broadly mirror the two key aims of this paper: [1] women’s personal experience with PNA; and [2] suggestions for online support (i.e. focusing on potential solutions). As such, the results section is broken down into two sections to reflect this:

1. Experiences with PNA: Sources of anxiety and sources of support

In terms of women’s experiences with PNA, 15 sub-themes were identified (see Table 2), which formed five major themes. The themes were grouped around two broad domains that mapped onto the study’s aims: understanding women’s sources of anxiety, and issues around support.

**Table 2:** Themes and sub-themes associated with women’s personal experience with PNA

Themes and Sub-Themes	Quotes
**SOURCES OF ANXIETY**	
**Unrealistic expectations of birth and motherhood**	
1. Expectations of childbirth	*FG5.F3: she did this demonstration about epidurals… and she made it seem just really scary… I came away being absolutely terrified of any intervention… But I ended up being induced and having forceps, both of which were… well the induction wasn’t fine… but the forceps were. So I felt there was a lot of misinformation about what an intervention would be like… which made me unnecessarily anxious.*
	*FG5.F2: my community midwife refused to tell me about induction because she thought it would happen naturally. But my waters broke before labour started, so I had to be induced… and I had no idea what was happening… (which) made me really frightened. And I remember thinking, but I’m allowed to ask you and you’re meant to tell me…*
2. Expectations of breastfeeding	*FG5.F3 It’s crazy how people make breastfeeding out to be this magical experience, when it just couldn’t be more different than that really.*
	*FG4.F2: [antenatal classes] show you this video of a five month old baby breastfeeding quite happily in some sort of coffee morning, and it’s absolutely easy. But then the reality is so different. And I felt just a bit tricked by the whole lead up to having a baby…*
3. Unrealistic guidelines and norms	*FG4.F2: It worries me when things like the Babycentre say like at eight months your child should be doing this, especially sleep and feeding… you feel like you’re just doing everything wrong.*
	*FG1.F3 I don’t know any mum who was able to put their child in a moses basket on their back from day one, and it just causes you an enormous amount of anxiety. So what are you meant to do, what can you do? I still don’t know.*
4. Unrealistic social comparison	*FG2.F2: I just struggle with parenthood being completely honest. It’s like one of these things where there’s so much social media these days saying how you should be this and that. And for me that’s actually, like the biggest thing that really blocks me from thinking actually it’s OK, like this is normal.*
5. Societal pressure	*FG1.F5: I think they put so much pressure on a mother by saying breast is best which means… yes it’s all positive. But… if you don’t succeed then… you’ve failed as a mother, you’ve failed your kids and it’s just not true because fed is the best isn’t it?*
	*FG1.F4: We’re still co-sleeping and everybody’s like… it’s so bad because SIDS and everything. Your babies will die if you co-sleep*
**Importance of Peer Support**	
6. Offline social support	*FG1.F6: Being here and away from family… has been the hardest part for me… but the girls in the (antenatal) group have been great. I think they’ve been the biggest support through all of this.*
7. Online peer support (positive and negative)	*FG5.F3: It definitely increases anxiety – those big forums definitely do. Because nobody wants to post when things go well. They just say terrible things…. which just made me really paranoid.*
	*FG4.F1: I found those (Facebook) groups just brilliantly good peer support, and more helpful than some of the breastfeeding groups I’ve been in.*
8. Normalisation of experience	*FG2, F1: So we just post whatever’s going on. So if you’re having a really naff day you’ll say Jesus Christ, the kids have done this, this and this. And someone goes oh tell me about it. And it’s just somebody, like, who’s feeling the same thing as you, so it’s not completely like… all the social media where it’s this perfect mum… it’s well I’m here with cereal stuck in my hair and it’s two o’clock in the afternoon. So at least with that it was other normal mums that were just like yeah, I’m doing the same thing as you. So you didn’t feel so alone.*
**Uncertainty and Maternal Confidence**	
9. Unprepared for change	*FG1.F1: I had absolutely no idea about any of motherhood I realised, y’know, the practical stuff.*
	*FG4.F2: That’s actually why people then struggle. I suppose we all go through our lives being good at a lot of things. You’ve established a career, you’ve established yourself, you’ve got qualifications. And then suddenly… this baby arrives and you’re not good at anything.*
10. Maternal confidence and overwhelm	*FG1, F4: I was overwhelmed with everything, the household running, and I only had to look after the baby. But it was so demanding.*
	*FG4, F1: (I have to know) when our daughter last fed, how much she’s fed and how much she’s vomited that day, how many wet nappies she’s had, and when her immunisation’s due… There’s this massive load that we’re carrying… And then you add to it just stupid things like the washing basket’s full…or I need to sterilise some things, and I need to do this and I need to do this. And I just find that there’s this constant like whir going on in my head.*
11. Conflicting (or extreme) information	*FG1, F1: I think (the internet) is a curse and a blessing. Because in the very early days you Google everything and there’s all these forums, there’s Netmums, there’s a blog, a Facebook thing that I was on Mummy’s Gin Fund… And the actual problem is there’s like too much advice and everyone’s – ‘do that’ and then there’ll be someone with complete opposite opinions saying do this, do that. And actually you get yourself into such a state because you almost read too much. And it made me very anxious, it didn’t really help that much…* *F2: …There’s just so much…* *F6: …and a lot of it’s extremes as well...* *F2: …Yeah… the last thing that I felt, or I feel that I need is like the polar opposite of opinions*
	*FG5, F2: What you read on the internet is quite extreme. And you always end up on message boards where one woman is like ‘I was induced and I was in labour for 72 h and then this happened to me’, and it just becomes really terrifying…*
**ISSUES AROUND SUPPORT**	
**Stigma**	
12. Internal and external stigma	*FG5.F4: I felt like I couldn’t talk to anyone about it, because I didn’t think it was normal and I felt like it was me not being a good mum* *F3: Me too. Yeah, exactly. I thought people might think I was an awful mother.*
13. Disclosure avoidance	*FG2.F2: I fear that if I went to somebody saying look I really suffer from anxiety, I get worried on who’s going to get involved with my kids.* *F1: That was my fear, that social services would get involved if anyone found out.*
**Lack of Mental Health Support and Knowledge**	
14. Lack of postnatal healthcare support	*FG5.F4: You’re all geared up to give birth, and then you do it and you get home and you’re like, shit, now I have a baby… and there’s no support really. When I had her, I had really bad anxiety, like postnatal anxiety full on. Like I couldn’t leave the house, I couldn’t leave her to sleep, like I couldn’t let her be put down, I was sleeping in like 2 h shifts because I was just terrified that she was going to die… And it got to the point where I had like a complete melt down and I didn’t know why. But no one spotted it. I’d done, I’d had my initial appointment with the midwife or health visitor, whoever it was, but it was so rushed. They didn’t get it. I had to contact my GP myself to ask, y’know, to be put on the mental health service lists… but y’know, nobody contacted me. I waited for like 3 weeks…*
15. Mental health literacy	*FG5.F2: I felt like I was going totally mad. I knew I didn’t have postnatal depression… and I did those questions with the health visitor, and she seemed to think I was ok. But then I couldn’t stop worrying about (the baby). I’d constantly check on her. And I didn’t want to go out in case something bad happened. And that’s not right. I didn’t know what it was, but I didn’t feel depressed.*
	*FG3, F2: I kept reading the books… and one page was on postnatal depression. I was like I haven’t got any of the symptoms, if anything I’m really happy but I’m not right.*
	*FG2.F1: it definitely helps just knowing what it is. Because once I knew what it was I could, it was calmer in my mind a little bit.*

### Unrealistic expectations of birth and motherhood

#### Expectations of childbirth

A consistent theme that emerged in all FGs was that women felt their expectations of the transition into motherhood were unrealistic, and often poorly managed. This was particularly evident in the context of antenatal classes, which women described as “very subjective” (FG5.F4) and having “a massive agenda” (FG5.F1). Some women felt that a ‘natural birth’ had been pushed on them, and that scaremongering tactics had been used to deter them from investigating more medicalised options. In some extreme cases, women felt almost brainwashed:

*FG5.F2: By the end of the sessions, I was like maybe I want a natural birth, I don’t want any pain relief. And someone said to me, why do you want those things? Is it because you really want those things, or is it just because you’ve been sort of conditioned to think that. And I didn’t know.*

In addition, those actively pursuing natural childbirth felt that there was an absence of realistic information (or sometimes misinformation) available about what to expect during labour, with some women reporting that their midwives actively withheld information about commonly occurring interventions, which often made them feel anxious, out of control and infantilised.

*FG5.F1: It’s almost like they treat you a bit like a child, like you can’t make your own decision so you can’t hear anything scary because you won’t be able to cope with it.*

#### Expectations of breastfeeding

A primary source of anxiety (and a topic that spontaneously arose in all FGs) was how unprepared women felt for the realities of breastfeeding. Many reported being “surprised”, “shocked” and “anxious” when they found breastfeeding to be “one of the hardest things I ever had to do.” (FG1.F5) and felt this was a direct result of the mismatch between their struggle in reality, and the information presented by antenatal classes, HCPs, social and mainstream media, which led them to expect breastfeeding would “be a magical experience”, “come naturally” or “be easy”.

Overall, women felt let down by the biased information they received about childbirth and breastfeeding, and expressed a need for more balanced and realistic information.

*FG5.F4: Why is this kind of bias allowed in a place where you know, you’re actually working with people who are quite vulnerable… people should be able to provide unbiased information about childbirth and breastfeeding.*

#### Unrealistic guidelines and norms

A further source of anxiety came from unrealistic guidelines and norms, for example the developmental milestones frequently reported on mum-focused websites (such as Bounty, BabyCentre etc). These resources aim to give parents a guide of what their child ‘should’ be doing at different ages. However, many of the mothers reported experiencing anxiety when their children did not show all of the skills on these lists.

*FG1.F5: (milestones) put pressure on you. Because you want to know, ok…why is my baby not doing this? … And then you start to Google if he doesn’t sit by this month what’s wrong with him?*

Many women also felt childcare guidelines were often “unrealistic” (particularly on the topics of sleep and feeding) and offered no alternative suggestions for situations when their babies did not adhere to the guidance advice.

#### Unrealistic social comparison

Social media was a particular source of anxiety, as it promoted unrealistic expectations of motherhood through social comparison. When faced with images of other mothers (either friends, family, celebrities or strangers) seemingly able to ‘bounce back’ immediately after childbirth, effortlessly managing to balance motherhood with other aspects of their life, and do everything ‘right’, many mothers reported experiencing intense feelings of failure and worry about their ability as a mother (see Table 2).

#### Societal pressure

Related to the previous sub-theme, women frequently stated that they felt under significant pressure to be the ‘perfect mum’ and to do ‘the right thing’ (which often equated to adhering to perceived societal norms, such as natural birth and breastfeeding); and felt judged by others on their mothering abilities and parenting choices. Furthermore, women frequently reported feeling an overwhelming sense of guilt and failure when they did not adhere to these societal scripts and fulfil the expectations they held about motherhood, often despite realising that these notions were unrealistic:

*FG4.F3: you do get a bit of rhetoric of this is the way that you should look after your baby, and then when it does deviate from that then you feel like you’re not meeting that standard.*

Throughout the quotes associated with this first theme was a sense that the women in this study felt they lacked agency, power and control in the perinatal period, often positioning themselves as novices in the situation and others as experts. For example, HCPs and antenatal class leaders were often characterised as gatekeepers to antenatal information, representing a lack of control or ability for the women themselves to make informed decisions about childbirth. Furthermore, discussions around milestones, guidelines and social comparisons often contained an implicit sense of powerlessness, as women both acknowledged that they were unrealistic, at the same time as aspiring to them. Societal scripts also gave way to a sense of helplessness and failure, with many women feeling that they did not live up to social expectations of what a good mother should be.

### Importance of peer support

#### Offline social support

Most women spoke about motherhood being isolating at times, which was a large source of distress. Having a supportive family and partner was described as being important and stress relieving, whilst an absence of support was often experienced as distressing. Participants consistently highlighted their peers as the single most important source of support. In most cases this was explicitly discussed in terms of face-to-face social support groups, such as antenatal classes (e.g. national childbirth trust, baby and bump, etc.) or breastfeeding cafes.

*FG5.F3: I would say this (antenatal group) has been the most important thing terms of support really.*

However, many of the FG participants did not take part in antenatal classes, primarily “*because it’s so expensive...”* (FG3.F1), which often led to feelings of isolation. Interestingly, this arose as an issue more in the rural FGs than the urban groups, and may be indicative of social-economic differences between the groups. In contrast to antenatal groups and breastfeeding groups, postnatal ‘mum and baby’ classes were not identified as an important source of support and were often experienced as isolating, as they do not encourage adult peer interaction (due to their primary focus on the babies).

*FG2.F1: I’d go to these classes… (but) people wouldn’t go by themselves, they’d all go in twos. And I’d sort of sit there and I’d be going I haven’t spoken to anyone.*

Furthermore, discussions suggested there were fewer mum-and-baby classes available in rural (compared to urban) settings. *(FG1.F5: if you live maybe in a small village, then I think you don’t have that much possibility to have these baby events.)* And some mothers reported being “*denied access to postnatal groups*” (FG2.F2) because they already had older children.

#### Online social support

Opinions of online support networks were somewhat mixed. Generally speaking, large scale forums were predominantly seen as anxiety-provoking, rather than relieving; while smaller online support groups (for example, Facebook groups with a single support focus, such as breastfeeding, pumping or gestational diabetes) were often characterised as being helpful. Indeed, participants often explicitly referred to this type of peer support as being more helpful than other, more professional, avenues of support.

*FG3.F2: Mumsnet gives me a little bit of anger…*

*FG3.F1: …Yeah, sometimes it makes you worry more.*

*FG2.F1: I would use (the Facebook group) for support there because I found, I tried the midwife, I tried the health visitor, I tried family and I tried the GP and I didn’t really get anywhere. So for me I used that because I don’t really… leave the house all that often... But actually I think the biggest support and the biggest help people get is other mums that are going through exactly the same thing.*

### Normalisation of experience

Whether the peer support was on- of off-line, the underlying mechanism of relief appeared to the same: the normalisation of their experience (i.e. the idea that is normal to deviate from the social ideals). Namely, women felt that they were “not alone” (FG1.F1), that “somebody else understands” (FG1.F5; FG5.F3) what they are going through, and that the struggles, uncertainty and chaos that parenthood brings with it is normal. Many women expressed a moment of clarity or explicit relief when they realised it was ok not to be coping perfectly; or to be living up to this fictional image of the “perfect mum”.

*FG1.F1: it was one mum… it was so refreshing, she said I didn’t really enjoy it until he was six months old. And something clicked in my brain then where I was like finally somebody… And suddenly just having someone say it took the pressure off and I could start to enjoy it (and) give myself a bit of a break*

### Uncertainty and maternal confidence

#### Unprepared for change

Many women described struggling with the adjustment to motherhood, whether as a first-time mother, or not. They felt unprepared for the demands of motherhood (often due to lack of knowledge and experience), which some women felt was the result of minimal exposure to the realities of motherhood, possibly as a result of poor inter-generational integration (in comparison to the past, or non-Western cultures) and a general lack of realistic life skills education*.*

*FG4.F3: There’s a gap where I didn’t know anything about children until I was 35… and I think that was really too late. And I think all of these issues around y’know… how does breastfeeding work… or how to deal with different labour scenarios. For me coming into that being new at 35 was really too late I think… I don’t know why we don’t talk about it from school onwards.*

#### Maternal confidence and overwhelm

In all FGs women alluded to an initial uncertainty about their maternal competence, with many suggesting that they felt out of their depth, and uncertain about their choices, feelings and behaviours.

*FG1.F6: People always just say just trust your instincts, you’re the mother you know best. But I don’t know. I don’t have any instincts because I don’t know about this situation. So I actually find that… to be really anxiety inducing.*

Women often described feeling “overwhelmed” (FG3.F2) and found it difficult to balance the demands of motherhood with those of everyday life. Conversations often fell into ‘before/after’ childbirth narratives, with mothers often feeling that they were no longer able to do the things they used to, including finding the time to look after themselves.

*FG3.F1: I never prioritise (looking after myself) anymore but I should… I just can’t stop enough… to do it.*

#### Conflicting (or extreme) information

Online sources of parenting information were often described as anxiety-provoking. The overabundance of information and polarising opinions online left women feeling confused, and not knowing who to trust (see Table 2). Confusing, conflicting information was also experienced in an offline context, with women frequently reporting receiving different guidance from HCPs. Mothers felt that these were usually just “people’s subjective opinions” and felt there wasn’t enough evidence-based, unbiased and/or middle-ground information to address their concerns.

*FG5.F3 “The midwife said one thing, the doctor said another, the two antenatal classes (I went to) gave exact opposite advice, and don’t even get me started on the internet. That just seemed to be one extreme or the other… So I was like, I don’t know what to do…. I don’t know who to listen to.”*

Interestingly, while women felt under pressure to live up to socially constructed ideals of motherhood, the presence of conflicting advice reveals how there is not really one true ideal. Not only is this confusing (and inherently contradictory), but also has the propensity to position women as always being wrong, regardless of what they do and what advice they follow.

### Stigma

#### Internal and external stigma

Several women reported feeling ashamed or “embarrassed” of their PNA symptoms, demonstrating internalised stigmatising attitudes directed towards themselves. This was usually accompanied by a demonstration of external stigma (i.e. having concerns about how others would see them if they told them about their anxiety). For example, many women spoke about deliberately hiding their symptoms from others, as they were worried that they would negatively judge them and/or their parenting abilities if they found out about their symptoms (see Table 2).

#### Disclosure avoidance

Disclosure avoidance related to women’s concerns about seeking professional help. Related to internal and external stigma, many women felt reluctant to disclose their symptoms to HCPs. This was largely due to a fear that they would be seen as a bad mother, and that there may be significant negative consequences as a result (for example, worrying that their baby may be taken away from them, or that social services might intervene). Thus, stigma acted as both a source of anxiety and as a barrier to help-seeking behaviours.

Apparent in both categories of stigma is a dichotomy that implies the notion that being a “good mother” is not compatible with mental illness; and having anxiety must therefore make you a “bad mother”.

### Lack of mental Health support and knowledge

#### Lack of HCP support

Overwhelmingly, women felt that while prenatal support from HCPs was generally good, there was very little support postnatally. Thus, the need for alternative avenues of support is paramount.

*FG1.F1: there hasn’t been a lot of help. Even if you go to the doctor about things like that… there doesn’t seem to be any groups or much information or resources.*

Several women felt they had “slipped through the net” and missed out on support, because they were not explicitly picked up as having an issue by their HCPs. Many felt that this was due to postnatal mental health screening procedures that are primarily focused on PND, and are not in-depth or maternally focused enough to pick up other mental health problems or indicative nuances in individual experience. This was likely compounded by aspects of stigma, which led some mothers to actively avoid disclosure.

#### Mental health literacy

Lack of knowledge about PNA and about maternal mental health was also an anxiety-inducing factor, and again acted as a barrier to seeking (or receiving) support. Many women reported feeling distressed about being unable to find any information about the way they were feeling either on- or off-line. In many cases they explicitly reported that they did not think that they had PND, but that they “*didn’t really know the difference between PND and anxiety*” (FG2.F1). These women said that they did not identify with PND symptoms, but were unable to find an alternative explanation of their symptoms which made them feel like they were “just going mad” (FG5.F3); *“I didn’t know what was wrong. I thought I’d just lost the plot.” FG3.F2.*

Several mothers reported feeling a significant sense of relief once they were able to identify that they probably had PNA (either via HCP intervention or self-diagnosis), highlighting the role of uncertainty in anxiety:

*FG5.F1: Once I knew that was happening it was easier… because I knew what to expect.*

And many mentioned that knowing what was wrong acted as a facilitator for access to support. However, even when it was recognised, some women felt there was a lack of information about how to cope with PNA, and what they could do to relieve symptoms.

*FG2.F1: For me I just haven’t found… coping strategies. As in if you’re having an anxiety attack it’s quite hard to have that when you’re looking after children… What’s the best thing to do when you have an anxiety attack?*

Overall, several common sources of anxiety were identified by the participants, and a number of issues around current support were highlighted.

(2) Suggestions for supporting PNA online

Discussions around women’s experience of and suggestions for online support identified 16 sub-themes (see Table 3), from which seven key themes emerged.

**Table 3:** Identified themes, subthemes and quotes related to supporting PNA online

Categories	Quotes
**Better preparation and management of expectations of birth and motherhood**	
1. Unbiased, balanced information	*FG5.F4: So maybe just like more information about what happens after you have the baby… like a little factsheet… you know, like there is clusterfeeding, things about sleep, because I think that’s why you get the anxiety isn’t it. Because you’re all geared up to give birth, and then you do it and you get home and you’re like, shit… now I have a baby… and you don’t know what’s normal or not.*
	*FG5.F1: Impartial advice. Which I know is really difficult, but just having facts for me would be really helpful. Like the reason people tell you to do stuff, rather than them just telling you to do it. So like the reason that we would tell you to do this is X, Y and Z… but if you didn’t want to do it then fine. I think it’s the way you use the language as well…*
2. Realistic information	*F5.F5: I just think that they need to give you realistic information for when your baby doesn’t fit into the guideline… like when they won’t sleep on their backs or whatever. Realistically, are you supposed to stand over them all night and turn them back over each time? That’s impossible.* *F2: So maybe some realistic advice would be good. Instead of it all being so black and white.*
	*FG1.F3: I think just balanced views and like also some realistic, I can’t think of what the word is but realistic experience or something.*
3. Evidenced-based information	*FG1.F3: KellyMom was quite good… because it’s not just everyone giving you their opinion. It’s hugely like y’know researched articles that they’ve got… In my experience that’s more balanced…*
4. Expert advice	*FG1.F2: If there would be a specialist… you can ask questions and that you can get support. I think that could, I think maybe allay a little bit of the anxiety because then you know that you can trust the expert’s advice and then you can relax because you feel like you’re on the right track.*
	*FG3.F2: If you could get like a list of all these worries that are quite common for mums, and then like some sort of answer to them. That would be really helpful wouldn’t it?*
**Peer support**	
5. Moderated peer input	*FG5.F1: There’s an app I use where people pose questions, and people answer them. But the answers are moderated and I think they pick like 5 or 6 answers that you see… You get like the expert answer and then you get like real mum’s answers. But the real mums answers, I think they pick them… ummm… moderate them. They’re generally quite like ummm… helpful. They’re not just like, ‘oh my god this happened to me and it was so awful…’ Which is good, because I don’t want to read someone’s nightmare story… that’s going to make me feel so much worse.*
	*FG5.F5: For breastfeeding, uhhh… there’s a UK Facebook support group. And that’s quite good, because the admins will turn off commenting. Or they will come in and say… you’re giving inappropriate advice here, this is what we would suggest. And I find that a bit more balanced.*
6. Limited peer input	*FG4.F5: One of my main sources of support is my NCT group because there’s eight of us, and that’s a nice number. It’s not too many voices and you feel like you can share and be open… so I don’t know if there is any scope to have an online version of that for people who can’t afford the NCT.*
7. Relevant adjunctive face-to-face support	*FG2.F2: When you go in a baby group it’s not like this… it’s not focused on the parents. I think it would be nice if you go to a baby group like this, where it’s focused on the whole idea of getting you all to talk to one another and something like that would be really helpful for someone like me.*
	*FG2.F1: If there was a postnatal depression or anxiety group where you could bring your kids but you could also chat to other people… that’d be good.*
	*FG3.F1: it would be good to have something where you could have a chat with the other mums…where the babies are happy but you can actually have a conversation as well with other… because sometimes you pay for those groups, and then you arrive. And the whole session is really busy, you don’t really talk to anyone else, and then you all go… even having 20 min after groups where people can get their bags together slowly, or there’s toys out where the babies can slowly leave, rather than everyone out the door. Because then you can actually make other contacts with people.*
**Normalisation**	
8. Normalisation of feelings through exposure to other mums	*F1.FG1: I saw something really good… it was so simple, they had three women sat around with a cup of tea in a kitchen and they just spoke to each other like in a conversation about their postnatal anxiety problems. And it was just really lovely because you just felt like you weren’t alone... So something like that where there’s a bit of video content, and again like, you know, for someone who hasn’t had a lot of sleep and can’t read a lot, that would be really good I think.*
	*FG2.F2: I think maybe if you had somewhere you could watch little video clips of people just talking openly with the kids in the background, or doing whatever. Maybe just little things like that…*
9. Humour as medicine	*FG1.F5: I ended up following a public figure who was writing a book The Unmumsy Mum and they just said like very silly things. So I just ended up reading a lot of silly things that she’s been doing with her kids that makes it OK not to be the perfect mum. And that helped me to let go of other stuff as well. So I think just making things funny, maybe sometimes a good laugh can release some of the negative energy as well.*
**Information about PNA symptoms and management**	
10. Information about PNA	*FG3.F2: On this internet programme I have to do (questionnaires) every single week… and yeah they’re quite helpful. Because the first time I did them… I was like oh so other people think that too.*
	*FG2.F1: Yeah, and also understanding what is actually wrong with you… And then I guess then once you know you’ve got something, or that something’s building up, you can, I read a lot about it and I understood a lot about it and it helped me, so maybe some information (about PNA) on a website.*
11. Anxiety management	*FG2.F1: Coping strategies… I’d find that quite useful... just how to manage it almost.*
**Increasing maternal confidence and wellbeing**	
12. General wellbeing support	*FG1.F1: Could it be about helping mums with their wellbeing as well…So maybe that wellbeing as well as factual like about breastfeeding advice and that. I would love to have some more information about things that you can do to look after yourself.*
	*FG1.F6: People always just say just trust your instincts, you’re the mother you know best. But I don’t know. I don’t have any instincts because I don’t know about this situation. So I actually find that… to be really anxiety inducing.*
	*FG3.F2: If somebody actually text me and say ‘think about you for 5 min… I might actually do it… you know’*
**Accessible to audience**	
13. Video and audio content	*FG1.F1: I don’t have a lot of time to read as well, so I don’t know if there could be elements where it’s audio. So I listened to a lot of podcasts and that was really helpful in the early days… I just think when you’re so sleep deprived. I don’t know about anyone else but completing a form or reading, like, a website full of paragraphs I couldn’t do it. So thinking about how you translate that information would be really helpful.*
	*FG3.F2: The only thing I can suggest is like… on You Tube, there’s some videos that you can watch… I could say to myself I’m going to have 10 min to watch this video, and it’ll be, I don’t know, just nice nature images and reaffirming things… something like that for mums postnatally to watch, that might be the way to just sit down and watch something. It would be nice when you can’t, you just haven’t got the energy to do it yourself.*
14. Easy to navigate	*FG1.F2: spend a lot of time on how the website looks, and how you navigate through it. And that will mean that people have the time and the inclination to use the resource, rather than it just to be too difficult, because when you’re sleep deprived and you don’t have time… y’know… I mean like easily structured and with headings and click routes on different categories of things.*
15. Easy to find	*FG1.F1: Make sure you make yourself visible because that is the hardest thing, is finding.*
**Privacy**	
16. Discreet	*FG2.F1: The only thing I find off putting… is if (websites) can post on my Facebook page… that’s the only thing that would annoy me, is that I don’t want posts on Facebook page about my (problems).*

### Better preparation/management of expectations of birth and motherhood (subthemes 1–5)

Women found that much of their anxiety stemmed from a lack of maternal confidence, which was often related to the uncertainty they felt as a result of the often unrealistic, confusing and inconsistent information they had been exposed to about major aspects of motherhood (see Table 2). To help counter this, women frequently stated they wanted access to *unbiased, balanced and realistic information* about important aspects of motherhood, namely labour/childbirth, breastfeeding and sleep management. Where possible, they wanted to see the *evidence-base* behind claims and guidelines, so they could understand their origins and enable them to make more informed choices about their actions. While they wanted *expert advice* on these matters, they also wanted access to realistic perspectives in the form of *moderated peer input* (limited to avoid the replication of problems seen in big forums, such as the presentation of too many opinions, and extreme opposite views).

### Peer support (subthemes 5–7)

The topic of peer support came up in all FGs. In particular, women wanted to hear other mother’s stories and experiences to help make sense of their own (see Theme 2). However, many mothers did not feel that this should be done in the form of a forum.

*FG5.F4: If there is going to be a forum, what is the purpose of the forum? And how… what role does it play in addition to what you want to offer. And if you are trying to offer you know, evidence-based information then I’m not sure it’s that helpful because it…*

*FG5.F3: …it muddies it…*

*FG5.F4: …yeah, yeah it does. And also I think Mumsnet… already does that job.*

Instead, women said they would prefer to hear from peers in a more moderated manner. And while the study aimed to elicit suggestions for supporting PNA online, all FGs suggested that internet-based support would greatly benefit from a face-to-face component. Interestingly, most women spontaneously commented on how supportive and useful they found the FGs themselves, with some women explicitly suggesting that the mum-focused nature of the interviews would be a useful model of support for future mum-and-baby classes:

*FG2.F2: When you go in a baby group it’s not like this… it’s not focused on the parents. I think it would be nice if you go to a baby group like this, where it’s focused on the whole idea of getting you all to talk to one another and something like that would be really helpful for someone like me.*

### Normalisation (subthemes 8–9)

An important theme that consistently emerged was that any online support should actively challenge the ‘perfect mum’ myth, so often propagated in society. Participants explicitly stated that they wanted to hear about other mothers' realistic experiences, to normalise their own. Many women reported finding humorous descriptions of everyday motherhood particularly relieving (such as The Unmumsy Mum, or Hurrah for Gin), and would like to see more of that represented online.

### Information about PNA symptoms and management (subthemes 10–11)

All FGs felt that future online support should have a psychoeducation element to it, enabling women to better identify their symptoms through the use of *diagnostic questionnaires* and by providing evidence-based information about PNA. In addition, they wanted to receive guidance on *anxiety management* either through advice on how to seek help, or by providing direct therapeutic advice and coping strategies.

### Increasing maternal confidence and wellbeing (subthemes 12)

In addition to providing PNA-specific support, many women felt it would be important to offer more *general wellbeing support*; fostering maternal confidence and supporting them to find a balance in their lives.

### Accessible to audience (subthemes 13–15)

A key consideration for online support was that its content should be *appropriate for the audience*. Specifically, women consistently outlined a preference for information that is easy to understand, uses limited written text, and is delivered via different media (such as video and audio). Keeping written content concise seemed important for an audience who is potentially short on time, and likely sleep deprived. Furthermore, to encourage engagement and sustained usage, websites would need to be both *easy to find* and *easy to navigate*.

### Privacy (subthemes 16)

Finally, women felt a key barrier to engagement with a PNA site would be a lack of privacy. Women suggested any site like this should be discreet and, in particular, should not be linked to their social media profiles.

## Discussion

This study qualitatively explored women’s experience of anxiety triggers and support preferences in the perinatal period. The broad aim of this study was to use a bottom-up qualitative approach to identify common sources of anxiety as potential targets for intervention, and provide a useful framework for the development of acceptable online programs aimed at supporting PNA. To this end, the discussion will compare study findings to previous research, and synthesise the main themes, exploring current issues around support and discussing how to overcome them; and drawing together the main targets that were identified and the acceptable online solutions that may be able to address them.

### Bridging the gap: A need for online support

To receive support for perinatal mental health issues, a need must first be identified. In the UK, HCPs are expected to assess women’s mental state as part of their pre- and postnatal care [49]. However, many mothers in this study felt these assessments were not particularly helpful, with their symptoms going largely unrecognised, and resulting in relatively little support. This is not surprising given the most commonly used screening measure in the UK is the Edinburgh Postnatal Depression Scale which was developed to screen for PND, and may not be sensitive enough to pick up anxiety-related symptoms [50–52]. Women reported feeling less supported in the postnatal period (compare to the prenatal period), which parallels previous work that has highlighted inattention, staff and facility shortages, and a general lack of postnatal information and breastfeeding support as particularly problematic [53–55]. This is particularly concerning, as Coates at al [39] highlighted that a perceived lack of support in the perinatal period may play a role in exacerbating postnatal distress. Thus, there is a need for mothers to feel more supported during this time. In the current climate of economic cuts and NHS staffing shortages, alternative cost-effective, efficacious and far-reaching methods of support (such as that offered online) are important to consider, for both the prevention and treatment of PNA. Furthermore, the internet is likely to be an acceptable source of support for this cohort, as all mothers in this study reported using the internet in some way during their pregnancy and following childbirth, to access information and support.

### Psychoeducation and evidence-based support

#### PNA-specific information and support

Many women in this study reported a lack of knowledge about PNA, which left them feeling distressed and confused about their symptoms, and acted as a barrier to seeking and receiving support. In line with research by Coates et al. [40], most women in this study did not identify with descriptions of PND, so had little sense of what was actually wrong; particularly as the majority of perinatal mental health information, screening and interventions focuses on depression. The uncertainty of their situation seemed to feed their anxiety, and thus should be a primary target for intervention.

Supporting this notion, women in this study suggested providing impartial, balanced and evidence-based information online may help to overcome some of these issues; and was a factor that mothers felt was important to include on any PNA support site. Furthermore, delivering accurate and clinically helpful information online may be a particularly acceptable method of support for people with stigmatised conditions as they can access it anonymously, thus circumventing possible negative outcomes associated with stigma [56–58]. In particular, providing reliable and valid information about PNA symptoms, risks, outcomes and treatment may help to increase awareness and knowledge about PNA (for both mothers and HCPs), allowing for better rates of diagnosis and enabling mothers to get the help they need. For example, future websites should consider including validated PNA-specific measures, such as the PASS [59], the PSAS [60], the PRAQ-R [24] and the PrAS [61], alongside evidence-based treatment information to encourage better recognition of PNA and facilitate more timely access to support, which in turn may prevent symptom perseverance and escalation.

Furthermore, several women described wanting access to therapeutic strategies to deal with PNA as and when it occurs. However, a previous review of available websites for PNA found that this type of information and support was generally lacking [34]. Despite this, a recent systematic review of the treatment of PNA concluded that cognitive behavioural therapy (CBT) is likely to be the most efficacious, safe and attractive treatment for pregnant and postpartum women [62], thus using electronic health (eHealth) technologies to deliver computerised CBT interventions is likely to be efficacious. Mindfulness techniques may also be beneficial [63]. To date, only one PNA-specific web-based treatment program has been evaluated via RCT [64]. Unfortunately, this program was deemed unacceptable in its current format, with high attrition rates. Thus, careful consideration about what women would like to see online needs to feature at the heart of any program development and design in order to maximise user engagement.

#### Increasing maternal confidence and general wellbeing

As well as wanting access to PNA-specific information and support, mothers in this study also wanted support for more general maternal confidence and wellbeing. As with previous work in this area [39, 43, 65], many mothers reported feeling ‘out of their depth’, and expressed a lack of confidence in their abilities. They often felt overwhelmed by their new role, and wanted support in terms of how to look after themselves more generally. As maternal wellbeing and self-efficacy are often inexorably linked [66, 67], addressing these issues together is likely to be helpful. Supporting maternal confidence is also likely to have a positive impact on both anxiety symptoms and infant behaviour, as previous research has found an association between high maternal anxiety, maternal self-efficacy and early regulatory problems in infants [68]. Given the potentially negative psychological impact of problematic crying, sleeping and feeding behaviours (areas that were all highlighted as particularly distressing in this study), confidence-based interventions may provide a dual route to positively impacting women’s mental health. Thus, integrating evidence-based psychological interventions that have been shown to increase wellbeing and confidence would be worth including in any online intervention developed for PNA.

### Addressing stigma and disclosure

Stigma was a key theme that acted as both a source of anxiety for women, and as a barrier to seeking and receiving help. Many felt ashamed of their symptoms, and were worried that they might be seen as a “bad mother” or that their child(ren) might be taken away from them as a result, reflecting previous work that has found that the stigma associated with perinatal mental illness can be a significant barrier to women disclosing and seeking help [21, 22]. This supported previous research by Moore, Ayers and Drey [21], who extended Goffman’s [69] theory of “spoiled identity” to conceptualise how some women who have perinatal mental illness (PMI) have a distorted identity as a mother. They conceptualised PMI stigma as having three factors; internal, external and treatment stigma; all of which could be identified in the current study. Internal stigma related to women's feelings of maternal inadequacy because they were experiencing symptoms of PMI. External stigma referred to the negative stigmatising beliefs women believed others held about women with PMI. Treatment stigma (similar to disclosure avoidance) referred to the discrimination women with PMI expected to experience if they disclosed their symptoms or diagnosis to others, including HCPs. This included the belief that there would be negative consequences of disclosure, such as loss of custodial rights. These factors in isolation, or more often combined for women experiencing PNA, are likely to both cause distress and inhibit help seeking behaviour and disclosure.

Feelings of stigma may also be propagated through the presence of unrealistic notions of motherhood; something that was a common source of distress in this study. As with previous research [65, 70], women in this study often reported feeling under an enormous amount of pressure to live up to the image of the ‘perfect mum’ so often portrayed by social (and mainstream) media, and expressed experiencing significant anxiety when they were unable to live up to these ideals. Therefore, future online support should seek to reconceptualise a new maternal identity for women with PNA. This identity would be positive and work on promoting more realistic expectations of motherhood, thus altering the standard women hold themselves to, moving from “perfect mum” to “good mum”. Websites have the potential to challenge internal stigma by reframing what a “good mum” is, extending the construct to include woman with PNA (and PMI more generally).

Similar to previous research, women in this study expressed an intertwining of external and disclosure stigma [21]. They worried that others would perceive them as a “bad mum” if they knew they had PNA and that if they disclosed to HCPs there would be negative consequences. Website content could challenge both external and disclosure stigma by exploring these barriers to care and normalising the stigmatised symptoms. Content could address their fears of professionals removing children from their care and promote more realistic and caring narratives, including information about how HCPs can support mothers to care for their child. This might be particularly effective through real stories of mothers who disclosed to non-judgemental professionals and recovered. Therefore, the concept of a “good mum” could be further extended to a mother with PNA who discloses and gets help.

### Managing expectations

In line with previous research [43, 71], findings from this study supported the notion that socially constructed myths of motherhood (including the idea that the transition to motherhood is an easy natural process, governed by automatic maternal instinct) can cause women to lose confidence and feel anxious when the reality of their experience does not match up to their expectations. In particular, mothers felt they had been inadequately prepared for birth and motherhood by their antenatal classes and (in some cases) HCPs, stating that their expectations of childbirth and motherhood had been poorly managed.

As with other studies that have explored perinatal distress in broader terms [39, 65], expectations and experiences of childbirth and breastfeeding were of particular concern. Women often reported feeling out of control in terms of their birthing choices and experiences, which in turn, caused them significant stress and anxiety. This mirrors previous work that has found a link between low perceived control, poor birth satisfaction and PND [72, 73]. Specifically, women felt birthing information was heavily biased towards natural birthing options, and that they were often discouraged from exploring (or were being protected from) more medicalised interventions, skewing women’s expectations of childbirth. A lack of access to realistic information about the reality of childbirth is likely to result in significant differences between expectations and reality, causing undue stress and anxiety. Thus, there is a need for realistic, rather than idealistic, childbirth preparation that gives inclusive information about the different forms childbirth may take. This is something that may be achieved online, and offers a possible avenue for PNA prevention strategies.

Similarly, the stark contrast between women’s experiences with breastfeeding and their notions of what it would be like were a significant source of worry. Comparable to work by Fox et al. [74], most women explicitly blamed this mismatch between reality and expectations on the information they received from their antenatal classes and HCPs, which suggested breastfeeding should be easy, painless and come naturally; effectively setting up a socially constructed idealised notion of what one of the central aspects of early motherhood should look like. Almost all women in the FGs reported struggling with breastfeeding at some point. They felt shocked at how difficult and relentless they found breastfeeding to be, and often reported feeling like a failure when it did not come easily to them, or anxious that something was wrong with them or their baby. To alleviate the stress and distress this caused, they often stopped breastfeeding altogether; echoing research that has found an association between postnatal anxiety and reduced breastfeeding durations [11, 75]. While many women in this study described actively wanting to breastfeed, they felt unsupported by HCPs and were uncertain where to find help. Those who found realistic and helpful information online or offline in Breastfeeding Support groups, reported feeling relief to learn that they were not alone in their struggle, which often helped them to continue breastfeeding and went some way towards ‘normalising’ their own experience. Again, providing realistic information about key aspects of motherhood should therefore be key targets for PNA prevention and support.

Additionally, women often reported feeling (unnecessarily) anxious when their child did not conform to certain externally shaped physiological, cognitive or behavioural expectations. In particular, women referred to weight goals, childcare guidance about how their baby ‘should’ be sleeping or feeding, and (more commonly) developmental milestones that appear online. This is understandable, as numerous mum-focused websites report these milestones and goals, feeding into an idealised construct of what ‘normal’ should look like. However, they usually lack any academic references and often fail to acknowledge the enormous natural variation in skill acquisition throughout the lifespan, causing women to worry unnecessarily when their child inevitably does not achieve all of these goals. Thus, there is a need for more transparent, evidence-based information around this; and statistical literacy about what falling into low centile weight categories, or missing suggested milestones actually means in real terms.

Overall, women explicitly reported feeling let down by the idealised images of childbirth and breastfeeding, and the lack of information about realistic experiences they were exposed to prenatally. This is a key area where women felt they could be better supported online. Participants repeatedly expressed a need for access to trustworthy, unbiased and balanced, realistic information about important aspects of motherhood. While they wanted some expert advice on these matters (which could be achieved in a variety of ways in an online environment), they particularly wanted to see authentic depictions of motherhood and solutions.

### Peer voices: ‘experts by experience’

The potential therapeutic value of hearing about other mothers’ experiences is a notion that cut cross many of the themes identified by this research. Peers were consistently identified as one of the most important sources of support for women with PNA, mirroring research that has found a link between social support and antenatal and postnatal depression [17, 76], and maternal self-efficacy and general wellbeing [66]. Furthermore, face-to-face peer-led interventions have been shown to have some success in reducing feelings of depression and isolation, and increasing confidence and self-esteem [77, 78].

However, while women wanted a degree of peer input online, they did not want to be overwhelmed by it, and they generally expressed a preference for a balance between expert and peer voices. Specifically, they felt unmoderated peer input (e.g. in large forums) was generally anxiety-provoking, while smaller topic-focused moderated forums (like Facebook groups) were deemed more useful. However, many mothers avoided participating in online forum discussions altogether, preferring to ‘lurk’ or passively engage with peer-led information (consistent with research that suggests up to 90% of forum users are lurkers [79]). Thus, to maximise inclusion and engagement, online methods of PNA support should move beyond forum-based models.

Contrary to findings by McLeish and Redshaw [80], whose interviews suggested that one of the most important therapeutic mechanisms of perinatal peer support was through being heard and understood, the current study found that it was the simple act of hearing about other mothers’ stories that provided participants with the most relief. Why this difference occurred is unclear, but it may be due to the different contexts of the two studies, with the first focussing on face-to-face interventions, and the current study being largely couched in online terms. In this case, participants suggested that hearing others’ experiences was helpful as it broke down unrealistic notions of motherhood, and often explicitly led to the realisation that they were not abnormal, inadequate or alone in their experience. This idea that normalising women’s experience of motherhood can lead to reduced PNA, maps onto previous research that has emphasised the role of appreciating shared human experience in reducing distress [39, 43], and is in line with work that has linked perfectionism and unattainable high standards to postnatal anxiety and distress [81, 82]. Furthermore, the importance of peer voices could potentially be couched in terms of Smith’s theoretical model of the relational self [83] which suggests the transition to motherhood involves the negotiation and emergence of a new identity (e.g. as a new mother, or mother to a new child), which is socially and relationally determined. Of particular relevance to this study’s findings is the idea that exposure to peers may serve as a “social-developmental preparation for motherhood”, giving mothers the opportunity to mentally rehearse important aspects of their roles through the observed experience of others. Smith posits that convergence between the self and others may be particularly important in terms of self-coherence, so the presentation of authentic experiences of others (that mothers can realistically relate to) may help to strengthen maternal identity and confidence. This theory may also go some way towards explaining why women in the current study found social depictions of idealised versions of motherhood (e.g. through social media) problematic; as they often represent caricatures that diverge from more realistic or ‘normal’ experience, which may have negative implications for maternal identity.

Using an ‘experts-by-experience’ model that uses peers with similar experiences to deliver mental health and support information has been shown to successfully challenge perceptions about mental illness, reduce stigma, lessen symptoms and increase help-seeking behaviours in other areas [84]. Thus, effective relief may be achieved by presenting authentic stories of other mothers’ experiences of PNA and (where appropriate) treatment. Using digital story-telling to achieve this may also help users make sense of their own experiences, support them to better recognise and articulate their own symptoms, and allow them to explore different coping and treatment strategies available [85]. However, it is important to be mindful about how to present realistic peer experiences online. Given that this study highlighted that exposure to (idealised) images of women online was instrumental in promoting women’s unrealistic expectations of motherhood, any peer representations of mothers’ experience need to be included in a sensitive, but realistic matter, and should aim to capture the diverse range of experiences that women in this period can have.

### Website features

In addition to discussing the general content of a potential PNA support site, women in this study had explicit suggestions and concerns about how such a site should operate. In particular, women felt that content had to be appropriate for a largely anxious, time-poor and sleep deprived audience. They felt that written text should be kept to a minimum, and suggested the use of video and audio would be beneficial. Interestingly, this is consistent with Ashford et al’s [64] evaluation of the iWaWa program, where women expressed a preference for more concise and shorter content. Furthermore, to encourage engagement and sustained usage, women stated that websites would need to be both easy to find and easy to navigate; issues that are often lacking in current PNA websites [34]. Women were also concerned about privacy and confidentiality; they wanted to be able to use the site without it being visible to others (e.g. on Facebook or other social media sites). While the subject of security did not explicitly come up in these FGs, it seems reasonable to extend this concern to ensure that if any data is collected by the site, that this should be encrypted and secure so that others are unable to access it.

### Limitations

While qualitative studies provide rich detail into participants’ experiences and perspectives, it is important to acknowledge that findings from this study may not be generalizable to all mothers who have experienced PNA. Women who took part in the focus groups were mostly white, educated, heterosexual, and in normative relationships with their child(ren)‘s father. While every effort was made to recruit women with a range of internet usage experience (by using both on- and offline recruitment strategies), participants were self-selected and the majority found the study through online adverts; thus active internet users may be over-represented, and may have more motivation to explore online support for PNA. Further research should aim to include women from a wider range of socio-demographic backgrounds, relationship statuses, and internet usage patterns, and may want to consider using online interview methods to broaden the demographic reach by reducing the logistical constraints associated with attending face-to-face focus groups. Additionally, no consideration was given to fathers in this study; something that future research should consider, as PNA is not limited to women [51].

## Conclusions

This study identified several key sources of anxiety for women in the perinatal period, and offered potential avenues to support these issues online. The internet has the potential to fill the support gap many women reported experiencing after childbirth. Providing professional, evidence-based support and psychoeducation (with sources of evidence clearly cited) is likely to go some way to alleviate the confusion and uncertainty women feel, increasing their knowledge and awareness of PNA, allowing for better identification of symptoms and therapeutic support strategies. Including evidence-based techniques to help alleviate PNA symptoms, and increase maternal confidence and general wellbeing (most likely using CCBT and mindfulness based activities) is also likely to be acceptable and beneficial. The management of women’s expectations of birth and motherhood is also likely to be key to supporting PNA. Women felt their expectations had been unrealistically shaped by societal scripts, social and mainstream media, and poorly managed by antenatal classes and HCPs. Presenting authentic stories of other women’s experiences, alongside balanced and realistic information about key events in motherhood (i.e. childbirth, sleeping, breastfeeding/feeding) is likely to ameliorate some of the anxiety currently experienced by mothers in the perinatal period through the normalisation of mothers’ experiences and by directly challenging the good mother/bad mother discourse that was so often evident in the FGs. Running across these solutions is the notion of empowering women with knowledge, equipping them with a sense of agency and control, and normalising their experience through the breakdown of socially constructed ideals of motherhood.

## Supplementary information

**Additional file 1.**

## Data Availability

Anonymised versions of the datasets used and analysed during the current study are available from the corresponding author on reasonable request.

## Abbreviations

CBT: Cognitive Behavioural Therapy
CCBT: Computerised Cognitive Behavioural Therapy
HCP: Healthcare professional
NCT: National Childbirth Trust
NHS: National Health Service (UK)
PASS: Perinatal Anxiety Screening Scale
PMI: Perinatal Mental Illness
PNA: Perinatal anxiety
PND: Postnatal depression
PRAQ-R: Pregnancy-Related Anxiety Questionnaire (Revised)
PrAS: Pregnancy-Related Anxiety Scale
PSAS: Postpartum Specific Anxiety Scale

## References

[1] Dennis C-L, Falah-Hassani K, Shiri R. Prevalence of antenatal and postnatal anxiety: Systematic review and meta-analysis. Br J Psychiatry [Internet]. 2017 [cited 2018 Dec 12];210(05):315–323. Available from: https://www.cambridge.org/core/product/identifier/S0007125000281361/type/journal_article. 10.1192/bjp.bp.116.187179 28302701

[2] Wisner KL, Sit DKY, McShea MC, Rizzo DM, Zoretich RA, Hughes CL, et al. Onset timing, thoughts of self-harm, and diagnoses in postpartum women with screen-positive depression findings. JAMA psychiatry [Internet]. 2013 [cited 2018 Dec 12];70(5):490–498. Available from: http://www.ncbi.nlm.nih.gov/pubmed/23487258. 10.1001/jamapsychiatry.2013.87 PMC4440326 23487258

[3] Bennett HA, Einarson A, Taddio A, Koren G, Einarson TR. Prevalence of Depression During Pregnancy: Systematic Review. Obstet Gynecol [Internet]. 2004 [cited 2018 Dec 13];103(4):698–709. Available from: http://www.ncbi.nlm.nih.gov/pubmed/15051562. 10.1097/01.AOG.0000116689.75396.5f 15051562

[4] Gavin NI, Gaynes BN, Lohr KN, Meltzer-Brody S, Gartlehner G, Swinson T. Perinatal Depression. Obstet Gynecol [Internet]. 2005 [cited 2018 Dec 12];106(5, part 1):1071–1083. Available from: http://www.ncbi.nlm.nih.gov/pubmed/16260528. 10.1097/01.AOG.0000183597.31630.db 16260528

[5] Wilson MC, Scior K (2014). Attitudes towards individuals with disabilities as measured by the implicit association test: A literature review. Res Dev Disabil [Internet].

[6] Ross LE, McLean LM. Anxiety disorders during pregnancy and the postpartum period: A systematic review. J Clin Psychiatry [Internet]. 2006 [cited 2018 Dec 13];67(8):1285–1298. Available from: http://www.ncbi.nlm.nih.gov/pubmed/16965210. 10.4088/jcp.v67n0818 16965210

[7] Fairbrother N, Janssen P, Antony MM, Tucker E, Young AH (2016). Perinatal anxiety disorder prevalence and incidence. J Affect Disord [Internet].

[8] Giardinelli L, Innocenti A, Benni L, Stefanini MC, Lino G, Lunardi C, et al. Depression and anxiety in perinatal period: prevalence and risk factors in an Italian sample. Arch Womens Ment Health [Internet]. 2012 [cited 2018 Dec 12];15(1):21–30. Available from: http://www.ncbi.nlm.nih.gov/pubmed/22205237. 10.1007/s00737-011-0249-8 22205237

[9] Glasheen C, Richardson GA, Fabio A (2010). A systematic review of the effects of postnatal maternal anxiety on children. Arch Womens Ment Health..

[10] Leach LS, Poyser C, Fairweather-Schmidt K (2017). Maternal perinatal anxiety: A review of prevalence and correlates. Clin Psychol.

[11] Paul IM, Downs DS, Schaefer EW, Beiler JS, Weisman CS (2013). Postpartum Anxiety and maternal-Infant Health outcomes. Pediatrics [Internet].

[12] Accortt EE, Wong MS (2017). It is time for routine screening for perinatal Mood and Anxiety Disorders in obstetrics and gynecology settings. Obstet Gynecol Surv.

[13] Howard LM, Molyneaux E, Dennis C-L, Rochat T, Stein A, Milgrom J. Series Perinatal mental health 1 Non-psychotic mental disorders in the perinatal period [Internet]. Vol. 384, www.thelancet.com. 2014 [cited 2018 Dec 12]. Available from: www.thelancet.com. 10.1016/S0140-6736(14)61276-9 25455248

[14] Gavin NI, Meltzer-Brody S, Glover V, Gaynes BN. Is Population-Based Identification of Perinatal Depression and Anxiety Desirable? Identifying Perinat Depress Anxiety [Internet]. 2015;(2015):11–31. Available from: http://doi.wiley.com/10.1002/9781118509722.ch1.

[15] Lonstein JS. Regulation of anxiety during the postpartum period. Front Neuroendocrinol [Internet]. 2007 [cited 2018 Dec 12];28(2–3):115–141. Available from: http://www.ncbi.nlm.nih.gov/pubmed/17604088. 10.1016/j.yfrne.2007.05.002 17604088

[16] Martini J, Petzoldt J, Einsle F, Beesdo-Baum K, Höfler M, Wittchen HU (2015). Risk factors and course patterns of anxiety and depressive disorders during pregnancy and after delivery: A prospective-longitudinal study. J Affect Disord.

[17] Leigh B, Milgrom J. Risk factors for antenatal depression, postnatal depression and parenting stress. BMC Psychiatry [Internet]. 2008 [cited 2019 may 29];8(1):24. Available from: http://www.ncbi.nlm.nih.gov/pubmed/18412979. 10.1186/1471-244X-8-24 PMC2375874 18412979

[18] O’Donnell KJ, Glover V, Barker ED, O’Connor TG. The persisting effect of maternal mood in pregnancy on childhood psychopathology. Dev Psychopathol [Internet]. 2014 [cited 2018 Dec 12];26(02):393–403. Available from: http://www.ncbi.nlm.nih.gov/pubmed/24621564. 10.1017/S0954579414000029 24621564

[19] Jorm AF, Kelly CM. Improving the public’s understanding and response to mental disorders. Aust Psychol [Internet]. 2007[cited 2019 May 29];42(2):81–89. Available from: http://doi.wiley.com/10.1080/00050060701280565.

[20] Maloni JA, Przeworski A, Damato EG. Web Recruitment and Internet Use and Preferences Reported by Women With Postpartum Depression After Pregnancy Complications. Arch Psychiatr Nurs [Internet]. 2013 [cited 2019 May 29];27(2):90–95. Available from: https://www.sciencedirect.com/science/article/abs/pii/S0883941712001653. 10.1016/j.apnu.2012.12.001 23540519

[21] Moore D, Ayers S, Drey N. A Thematic Analysis of Stigma and Disclosure for Perinatal Depression on an Online Forum. JMIR Ment Heal [Internet]. 2016 [cited 2018 Dec 12];3(2):e18. Available from: http://mental.jmir.org/2016/2/e18/. 10.2196/mental.5611 PMC4909386 27197516

[22] Bilszta, Justin; Ericksen, Jennifer; Buist, Anne; Milgrom J. Women’s Experience of Postnatal Depression - Beliefs and Attitudes as Barriers to Care. Aust J Adv Nursing [Internet]. 2010 [cited 2019 May 29];27(3):44–54. Available from: https://search.informit.com.au/documentSummary;dn=909929185761432;res=IELAPA.

[23] American Psychiatric Association (2013). Diagnostic and statistical manual of mental disorders (DSM-V).

[24] Huizink AC, Mulder EJH, Robles de Medina PG, Visser GHA, Buitelaar JK. Is pregnancy anxiety a distinctive syndrome? Early Hum Dev [Internet]. 2004 [cited 2018 Dec 12];79(2):81–91. Available from: https://www.sciencedirect.com/science/article/abs/pii/S037837820400074X. 10.1016/j.earlhumdev.2004.04.014 15324989

[25] Phillips J, Sharpe L, Matthey S, Charles M. Maternally focused worry. Arch Womens Ment Health [Internet]. 2009 [cited 2018 Dec 12];12(6):409–18. Available from: http://link.springer.com/10.1007/s00737-009-0091-4. 10.1007/s00737-009-0091-4 19626414

[26] Woolhouse H, Brown S, Krastev A, Perlen S, Gunn J. Seeking help for anxiety and depression after childbirth: results of the Maternal Health Study. Arch Womens Ment Health [Internet]. 2009 [cited 2019 may 29];12(2):75–83. Available from: http://www.ncbi.nlm.nih.gov/pubmed/19214705. 10.1007/s00737-009-0049-6 19214705

[27] Bauer A, Parsonage M, Knapp M, Iemmi V, Adelaja B. Costs of perinatal mental health problems. 2014 [cited 2019 May 29]; Available from: http://eprints.lse.ac.uk/59885/.

[28] Harrison V, Proudfoot J, Wee PP, Parker G, Pavlovic DH, Manicavasagar V (2011). Mobile mental health: Review of the emerging field and proof of concept study. J Ment Heal.

[29] Barak A, Hen L, Boniel-Nissim M, Shapira N. A Comprehensive Review and a Meta-Analysis of the Effectiveness of Internet-Based Psychotherapeutic Interventions. J Technol Hum Serv [Internet]. 2008 [cited 2019 May 29];26(2–4):109–60. Available from: http://www.tandfonline.com/doi/abs/10.1080/15228830802094429.

[30] Cuijpers P, Marks IM, van Straten A, Cavanagh K, Gega L, Andersson G. Computer-Aided Psychotherapy for Anxiety Disorders: A Meta-Analytic Review. Cogn Behav Ther [Internet]. 2009 [cited 2019 may 29];38(2):66–82. Available from: http://www.ncbi.nlm.nih.gov/pubmed/20183688. 10.1080/16506070802694776 20183688

[31] Griffiths KM, Christensen H. Review of randomised controlled trials of Internet interventions for mental disorders and related conditions. Clin Psychol [Internet]. 2006 [cited 2019 May 29];10(1):16–29. Available from: http://doi.wiley.com/10.1080/13284200500378696.

[32] Ashford MT, Ayers S, Olander EK (2017). Supporting women with postpartum anxiety: exploring views and experiences of specialist community public health nurses in the UK. Heal Soc Care Community.

[33] Moore D, Ayers S. A review of postnatal mental health websites: help for healthcare professionals and patients. Arch Womens Ment Health [Internet]. 2011 [cited 2018 Dec 12];14(6):443–452. Available from: http://www.ncbi.nlm.nih.gov/pubmed/22109827. 10.1007/s00737-011-0245-z 22109827

[34] Moore D, Harrison V. A review of websites for perinatal anxiety: Advice for healthcare professionals and users (Preprint). JMIR Ment Heal [Internet]. 2018; Available from: http://preprints.jmir.org/preprint/11464/accepted. 10.2196/11464 PMC6320400 30573444

[35] Danaher BG, Milgrom J, Seeley JR, Stuart S, Schembri C, Tyler MS, et al. MomMoodBooster Web-Based Intervention for Postpartum Depression: Feasibility Trial Results. J Med Internet Res [Internet]. 2013 [cited 2019 may 29];15(11):e242. Available from: http://www.ncbi.nlm.nih.gov/pubmed/24191345. 10.2196/jmir.2876 PMC3841354 24191345

[36] O’Mahen HA, Richards DA, Woodford J, Wilkinson E, McGinley J, Taylor RS, et al. Netmums: a phase II randomized controlled trial of a guided Internet behavioural activation treatment for postpartum depression. Psychol Med [Internet]. 2014 [cited 2019 may 29];44(8):1675–1689. Available from: http://www.ncbi.nlm.nih.gov/pubmed/24148703. 10.1017/S0033291713002092 PMC4005035 24148703

[37] Ashford MT, Olander EK, Ayers S. Computer- or web-based interventions for perinatal mental health: A systematic review. J Affect Disord [Internet]. 2016;197:134–46. Available from: 10.1016/j.jad.2016.02.057. 10.1016/j.jad.2016.02.057 26991368

[38] Biagianti B, Hidalgo-Mazzei D, Meyer N. Developing digital interventions for people living with serious mental illness: perspectives from three mHealth studies. Evid Based Ment Health [Internet]. 2017 [cited 2019 Feb 13s];20(4):98–101. Available from: http://www.ncbi.nlm.nih.gov/pubmed/29025862. 10.1136/eb-2017-102765 PMC5750413 29025862

[39] Coates R, Ayers S, de Visser R. Women’s experiences of postnatal distress: a qualitative study. BMC Pregnancy Childbirth [Internet]. 2014 14 [cited 2018 Dec 12];14(1):359. Available from: http://bmcpregnancychildbirth.biomedcentral.com/articles/10.1186/1471-2393-14-359. 10.1186/1471-2393-14-359 PMC4288655 25315742

[40] Coates R, de Visser R, Ayers S. Not identifying with postnatal depression: a qualitative study of women’s postnatal symptoms of distress and need for support. J Psychosom Obstet Gynecol [Internet]. 2015 [cited 2018 Dec 12];36(3):114–21. Available from: http://www.ncbi.nlm.nih.gov/pubmed/26135567. 10.3109/0167482X.2015.1059418 26135567

[41] Law KH, Jackson B, Guelfi K, Nguyen T, Dimmock JA. Understanding and alleviating maternal postpartum distress: Perspectives from first-time mothers in Australia. Soc Sci Med [Internet]. 2018 [cited 2019 Jun 28];204:59–66. Available from: https://www.sciencedirect.com/science/article/abs/pii/S0277953618301266. 10.1016/j.socscimed.2018.03.022 29579481

[42] Fallon V, Halford JCG, Bennett KM, Harrold JA (2016). The Postpartum specific Anxiety scale: development and preliminary validation. Arch Womens Ment Health [Internet].

[43] Wardrop AA, Popadiuk NE. The Qualitative Report Women’ s Experiences with Postpartum Anxiety: Expectations, Relationships, and Sociocultural Influences Recommended APA Citation. Qual Rep [Internet]. 2013 [cited 2018 Dec 13];18(3):1–21. Available from: https://nsuworks.nova.edu/tqr/vol18/iss3/2.

[44] Tausch AP, Menold N. Methodological Aspects of Focus Groups in Health Research: Results of Qualitative Interviews With Focus Group Moderators. Glob Qual Nurs Res [Internet]. 2016 [cited 2018 Dec 20];3:2333393616630466. Available from: http://www.ncbi.nlm.nih.gov/pubmed/28462326. 10.1177/2333393616630466 PMC5342644 28462326

[45] Kitzinger J. Qualitative research. Introducing focus groups. BMJ [Internet]. 1995 [cited 2019 may 29];311(7000):299–302. Available from: http://www.ncbi.nlm.nih.gov/pubmed/7633241. 10.1136/bmj.311.7000.299 PMC2550365 7633241

[46] Halcomb EJ, Gholizadeh L, DiGiacomo M, Phillips J, Davidson PM. Literature review: considerations in undertaking focus group research with culturally and linguistically diverse groups. J Clin Nurs [Internet]. 2007 [cited 2018 Dec 20];16(6):1000–11. Available from: http://www.ncbi.nlm.nih.gov/pubmed/17518876. 10.1111/j.1365-2702.2006.01760.x 17518876

[47] Ayers S, Coates R, Matthey S. Identifying Perinatal Anxiety. In: Identifying Perinatal Depression and Anxiety [Internet]. Chichester, UK: John Wiley & Sons, Ltd; 2015 [cited 2018 Dec 12]. p. 93–107. Available from: http://doi.wiley.com/10.1002/9781118509722.ch6.

[48] Braun V, Clarke V. Using thematic analysis in psychology. Qual Res Psychol [Internet]. 2006 [cited 2019 May 29];3(2):77–101. Available from: http://www.tandfonline.com/doi/abs/10.1191/1478088706qp063oa.

[49] National Institute for Health and Care Excellence. Antenatal and postnatal mental health: clinical management and service guidance [Internet]. London: NICE; 2014 [cited 2019 May 29]. Available from: https://www.nice.org.uk/guidance/cg192. 31990493

[50] Cox J, Holden J, Sagovsky R (1987). Detection of postnatal depression: development of the 10-item Edinburgh postnatal Depression scale. Br J Psychiatry.

[51] Matthey S, Barnett B, Howie P, Kavanagh DJ. Diagnosing postpartum depression in mothers and fathers: whatever happened to anxiety? J Affect Disord [Internet]. 2003 [cited 2019 29];74(2):139–47. Available from: https://www.sciencedirect.com/science/article/abs/pii/S0165032702000125. 10.1016/s0165-0327(02)00012-5 12706515

[52] Meades R, Ayers S (2011). Anxiety measures validated in perinatal populations: A systematic review. J Affect Disord.

[53] Beake S, McCourt C, Bick D. Women’s views of hospital and community-based postnatal care: the good, the bad and the indifferent. Evid Based Midwifery [Internet]. 2005 [cited 2019 ];3(2):80–6. Available from: https://repository.uwl.ac.uk/id/eprint/118/.

[54] Brown SJ, Davey M-A, Bruinsma FJ. Women’s views and experiences of postnatal hospital care in the Victorian Survey of Recent Mothers 2000. Midwifery [Internet]. 2005 [cited 2019 Feb 28];21(2):109–26. Available from: http://www.ncbi.nlm.nih.gov/pubmed/15878426. 10.1016/j.midw.2004.09.006 15878426

[55] Rudman A, Waldenström U. Critical views on postpartum care expressed by new mothers. BMC Health Serv Res [Internet]. 2007 [cited 2019 Feb 28];7(1):178. Available from: https://bmchealthservres.biomedcentral.com/articles/10.1186/1472-6963-7-178. 10.1186/1472-6963-7-178 PMC2216017 17983479

[56] Button S, Thornton A, Lee S, Shakespeare J, Ayers S. Seeking help for perinatal psychological distress: a meta-synthesis of women’s experiences. Br J Gen Pract [Internet]. 2017;bjgp17X692549. Available from: http://bjgp.org/lookup/doi/10.3399/bjgp17X692549. 10.3399/bjgp17X692549 PMC5604833 28847773

[57] Moore D, Drey N, Ayers S. Use of Online Forums for Perinatal Mental Illness, Stigma, and Disclosure: An Exploratory Model. JMIR Ment Heal [Internet]. 2017 [cited 2018 Dec 12];4(1):e6. Available from: http://mental.jmir.org/2017/1/e6/. 10.2196/mental.5926 PMC5339438 28219879

[58] Rains SA. The implications of stigma and anonymity for self-disclosure in health blogs. Health Commun [Internet]. 2014 [cited 2018 Dec 12];29(1):23–31. Available from: http://www.ncbi.nlm.nih.gov/pubmed/23356432. 10.1080/10410236.2012.714861 23356432

[59] Somerville S, Dedman K, Hagan R, Oxnam E, Wettinger M, Byrne S (2014). The perinatal Anxiety screening scale: development and preliminary validation. Arch Womens Ment Health..

[60] Fallon V, Halford JCG, Bennett KM, Harrold JA (2018). Postpartum-specific anxiety as a predictor of infant-feeding outcomes and perceptions of infant-feeding behaviours: new evidence for childbearing specific measures of mood. Arch Womens Ment Health..

[61] Brunton RJ, Dryer R, Krägeloh C, Saliba A, Kohlhoff J, Medvedev O. The pregnancy-related anxiety scale: A validity examination using Rasch analysis. J Affect Disord [Internet]. 2018 [cited 2019 Jun 28];236:127–35. Available from: http://www.ncbi.nlm.nih.gov/pubmed/29730512. 10.1016/j.jad.2018.04.116 29730512

[62] Marchesi C, Ossola P, Amerio A, Daniel BD, Tonna M, De Panfilis C (2016). Clinical management of perinatal anxiety disorders: A systematic review. J Affect Disord.

[63] Goodman JH, Guarino A, Chenausky K, Klein L, Prager J, Petersen R (2014). CALM pregnancy: results of a pilot study of mindfulness-based cognitive therapy for perinatal anxiety. Arch Womens Ment Health.

[64] Ashford MT, Olander EK, Rowe H, Fisher JR, Ayers S. Feasibility and Acceptability of a Web-Based Treatment with Telephone Support for Postpartum Women With Anxiety: Randomized Controlled Trial. JMIR Ment Heal [Internet]. 2018 [cited 2019 Mar 4];5(2):e19. Available from: http://mental.jmir.org/2018/2/e19/. 10.2196/mental.9106 PMC5938691 29678804

[65] Law KH, Jackson B, Guelfi K, Nguyen T, Dimmock JA (2018). Understanding and alleviating maternal postpartum distress: perspectives from first-time mothers in Australia. Soc Sci Med.

[66] Brazeau N, Reisz S, Jacobvitz D, George C. UNDERSTANDING THE CONNECTION BETWEEN ATTACHMENT TRAUMA AND MATERNAL SELF-EFFICACY IN DEPRESSED MOTHERS. Infant Ment Health J [Internet]. 2018 [cited 2019 Feb 25];39(1):30–43. Available from: http://www.ncbi.nlm.nih.gov/pubmed/29281747. 10.1002/imhj.21692 PMC5814850 29281747

[67] Carlsson I-M, Ziegert K, Nissen E. The relationship between childbirth self-efficacy and aspects of well-being, birth interventions and birth outcomes. Midwifery [Internet]. 2015 [cited 2019 Mar 4];31(10):1000–1007. Available from: https://www.sciencedirect.com/science/article/pii/S0266613815001539. 10.1016/j.midw.2015.05.005 26094071

[68] Matthies LM, Wallwiener S, Müller M, Doster A, Plewniok K, Feller S, et al. Maternal self-confidence during the first four months postpartum and its association with anxiety and early infant regulatory problems. Infant Behav Dev [Internet]. 2017 [cited 2019 Mar 4];49:228–22s. Available from: https://linkinghub.elsevier.com/retrieve/pii/S0163638317300620. 10.1016/j.infbeh.2017.09.011 28987983

[69] Goffman E. Stigma: notes on the management of spoiled identity. Englewood Cliffs: Prentice-Hall; 1963.

[70] Djafarova E, Trofimenko O. Exploring the relationships between self-presentation and self-esteem of mothers in social media in Russia. Comput Human Behav [Internet]. 2017 [cited 2019 Feb 18];73:20–27. Available from: https://linkinghub.elsevier.com/retrieve/pii/S074756321730170X.

[71] Lazarus K, Rossouw PJ (2015). Mother’s expectations of parenthood: The impact of prenatal expectations on self-esteem, depression, anxiety, and stress post birth. Int J Neuropsychother.

[72] Goodman P, Mackey MC, Tavakoli AS. Factors related to childbirth satisfaction. J Adv Nurs [Internet]. 2004 [cited 2019 May 29];46(2):212–219. Available from: http://doi.wiley.com/10.1111/j.1365-2648.2003.02981.x. 10.1111/j.1365-2648.2003.02981.x 15056335

[73] Soet JE, Brack GA, DiIorio C. Prevalence and Predictors of Women’s Experience of Psychological Trauma During Childbirth. Birth [Internet]. 2003 1 [cited 2019 May 29];30(1):36–46. Available from: http://doi.wiley.com/10.1046/j.1523-536X.2003.00215.x. 10.1046/j.1523-536x.2003.00215.x 12581038

[74] Fox R, McMullen S, Newburn M. UK women’s experiences of breastfeeding and additional breastfeeding support: a qualitative study of Baby Café services. BMC Pregnancy Childbirth [Internet]. 2015 [cited 2019 may 29];15:147. Available from: http://www.ncbi.nlm.nih.gov/pubmed/26148545. 10.1186/s12884-015-0581-5 PMC4494694 26148545

[75] Stuebe AM, Grewen K, Meltzer-Brody S. Association Between Maternal Mood and Oxytocin Response to Breastfeeding. J Women’s Heal [Internet]. 2013 Apr [cited 2019 may 29];22(4):352–61. Available from: http://www.ncbi.nlm.nih.gov/pubmed/23586800. 10.1089/jwh.2012.3768 PMC3627433 23586800

[76] Collins NL, Dunkel-Schetter C, Lobel M, Scrimshaw SC. Social support in pregnancy: psychosocial correlates of birth outcomes and postpartum depression. J Pers Soc Psychol [Internet]. 1993 [cited 2019 may 29];65(6):1243–58. Available from: http://www.ncbi.nlm.nih.gov/pubmed/8295121. 10.1037//0022-3514.65.6.1243 8295121

[77] Dennis C-L, Hodnett E, Kenton L, Weston J, Zupancic J, Stewart DE, et al. Effect of peer support on prevention of postnatal depression among high risk women: multisite randomised controlled trial. BMJ [Internet]. 2009 [cited 2019 may 29];338:a3064. Available from: http://www.ncbi.nlm.nih.gov/pubmed/19147637. 10.1136/bmj.a3064 PMC2628301 19147637

[78] Cupples ME, Stewart MC, Percy A, Hepper P, Murphy C, Halliday HL. A RCT of peer-mentoring for first-time mothers in socially disadvantaged areas (The MOMENTS Study). Arch Dis Child [Internet]. 2011 [cited 2019 may 29];96(3):252–8. Available from: http://www.ncbi.nlm.nih.gov/pubmed/20522466. 10.1136/adc.2009.167387 20522466

[79] van Mierlo T. The 1% rule in four digital health social networks: an observational study. J Med Internet Res [Internet]. 2014 [cited 2019 Mar 4];16(2):e33. Available from: http://www.jmir.org/2014/2/e33/. 10.2196/jmir.2966 PMC3939180 24496109

[80] McLeish J, Redshaw M. Mothers’ accounts of the impact on emotional wellbeing of organised peer support in pregnancy and early parenthood: a qualitative study. BMC Pregnancy Childbirth [Internet]. 2017 [cited 2019 Mar 4];17(1):28. Available from: http://bmcpregnancychildbirth.biomedcentral.com/articles/10.1186/s12884-017-1220-0. 10.1186/s12884-017-1220-0 PMC5237175 28086827

[81] Macedo A, Bos SC, Marques M, Maia B, Soares MJ, Pereira T, et al. Perfectionism dimensions in pregnancy—a study in Portuguese women. Arch Womens Ment Health [Internet]. 2009 [cited 2019 May 29];12(1):43–52. Available from: http://link.springer.com/10.1007/s00737-008-0042-5. 10.1007/s00737-008-0042-5 19159067

[82] Oddo-Sommerfeld S, Hain S, Louwen F, Schermelleh-Engel K. Longitudinal effects of dysfunctional perfectionism and avoidant personality style on postpartum mental disorders: Pathways through antepartum depression and anxiety. J Affect Disord [Internet]. 2016 [cited 2019 May 29];191:280–288. Available from: https://www.sciencedirect.com/science/article/abs/pii/S0165032715302731. 10.1016/j.jad.2015.11.040 26688497

[83] Smith JA. Identity development during the transition to motherhood: An interpretative phenomenological analysis. J Reprod Infant Psychol [Internet]. 1999 [cited 2019 Jun 28];17(3):281–299. Available from: http://www.tandfonline.com/doi/abs/10.1080/02646839908404595.

[84] Davidson T, Moreland A, Bunnell BE, Winkelmann J, Hamblen JL, Ruggiero KJ. Reducing Stigma in Mental Health Through Digital Storytelling. In: Canfield BA, Cunningham HA, editors. Deconstructing Stigma in Mental Health [Internet]. 2018 [cited 2019 May 29]. p. 169–83. Available from: http://services.igi-global.com/resolvedoi/resolve.aspx?doi=10.4018/978-1-5225-3808-0.ch007.

[85] De Vecchi N, Kenny A, Dickson-Swift V, Kidd S. How digital storytelling is used in mental health: A scoping review. Int J Ment Health Nurs [Internet]. 2016 [cited 2019 May 29];25(3):183–93. Available from: http://doi.wiley.com/10.1111/inm.12206. 10.1111/inm.12206 26900000

[86] Harrison V, Moore D. Supporting perinatal anxiety in the digital age: an exploration of stressors and support. In: Abstracts of papers and posters presented at 39th Annual SRIP Conference. 2019 5-6; City University of London. Journal of Reproductive and Infant Psychology; 2019 37(5), e1-e48, DOI: 10.1080/02646838.2019.1673570.

