# Supplementary material for: Supporting perinatal anxiety in the digital age; a qualitative exploration of stressors and support strategies
Source: BMC Pregnancy Childbirth. 2020 Jun 17;20:363. doi: 10.1186/s12884-020-02990-0 (PMC7298791; doi:10.1186/s12884-020-02990-0)
Supplement: Supplementary file 1 — Additional file 1. [file 12884_2020_2990_MOESM1_ESM.docx]

**Semi-Structured Interview Questions**

**Introduction**

Give background information about the study; introduce facilitators and start to gain a rapport with participants.

**Questions**

Allow 30 minutes

- Just as an opener, could you tell us your name and a little bit about your experiences with pregnancy or motherhood so far?
- We know that worry, anxiety and stress are very common in the period surrounding childbirth, so we’re interested to hear about women’s experiences with this…
  - What do you think are particular sources of anxiety for women in the perinatal period?

Allow 30 minutes

- We want to understand more about how women might go about seeking information, help and support for perinatal anxiety. If you were in that position, what kind of information and support do you think you might be looking for (if any)?
  - How do you think you might go about finding this?
- Would you consider using technology (like a website or app) to support you?
  - What do you think about this kind of support for women who are pregnant or new mothers?
- Have you ever used a mental health or wellbeing app or website?
  - What specific features of the app(s) impressed you?
  - What features of the app(s) disappointed or annoyed you?
  - What would make you decide to actually use this type of app/website?
- What would your dream app/website do? (Imagine there are no limits)
  - Of all the things we’ve just talked about, what is most important to you?

Additional Probe Examples: Could you explain further? Would you give me an example of what you mean? Would you say more? Is there anything else?
